# Supplementary material for: Idiotype-specific CD4+ T cells chronically stimulate autoreactive B cells to develop into B lymphomas in mice
Source: Nat Commun. 2026 Feb 25;17:3200. doi: 10.1038/s41467-026-69916-w (PMC13057116; doi:10.1038/s41467-026-69916-w)
Supplement: Supplementary file 1 — Supplementary Information [file 41467_2026_69916_MOESM1_ESM.pdf]

**Supplementary Information**

**Idiotype-specific CD4$^+$ T cells chronically stimulate autoreactive B cells to develop into B lymphomas in mice.**

Ramakrishna Prabhu Gopalakrishnan[1,2,*], Jerrold M. Ward[3], Victor Greiff[1,2], Ranveig Braathen[1,2], Xian Hu[4], Livia Bajelan[1], Khang Lê Quý[1,2], Ludvig Munthe[1,5], Peter Csaba Huszthy[2,†], Bjarne Bogen[1,2,*].

[1]Department of Immunology and Transfusion Medicine, Division of Laboratory Medicine, Oslo University Hospital, 0424 Oslo, Norway

[2]Department of Immunology, Institute of Clinical Medicine, University of Oslo, 0318 Oslo, Norway

[3]National Cancer Institute, NIH, Bethesda, MD USA 20892

[4]Center for Cancer Cell Reprogramming, Faculty of Medicine, University of Oslo, Oslo, Norway

[5]KG Jebsen Centre for B cell Malignancies and Precision Immunotherapy Alliance, University of Oslo, Oslo, Norway

[†]Current address: Department of Microbiology and Infection Control, Akershus University Hospital, 1478 Lørenskog, Norway

[*]Corresponding author: bjarne.bogen@medisin.uio.no

[*]Corresponding author: r.p.gopalakrishnan@medisin.uio.no

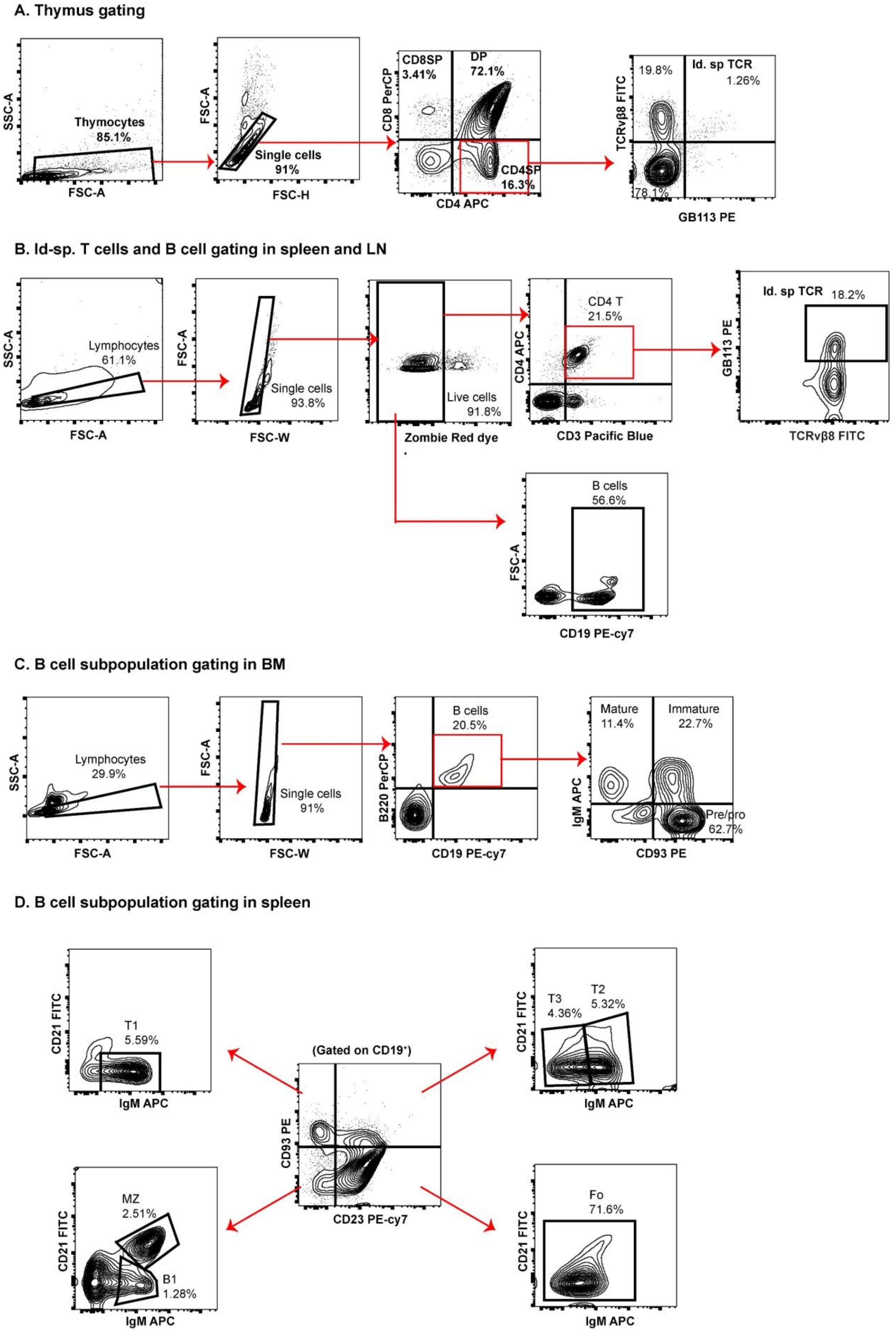
A. Thymus gating
SSC-A
FSC-A
Thymocytes
85.1%
FSC-A
FSC-H
Single cells
91%
CD8 PerCP
CD4 APC
CD8SP
3.41%
DP
72.1%
CD4SP
16.3%
TCRvβ8 FITC
GB113 PE
19.8%
Id. sp TCR
1.26%
78.1%
B. Id-sp. T cells and B cell gating in spleen and LN
SSC-A
FSC-A
Lymphocytes
61.1%
FSC-A
FSC-W
Single cells
93.8%
Zombie Red dye
Live cells
91.8%
CD4 APC
CD3 Pacific Blue
CD4 T
21.5%
GB113 PE
TCRvβ8 FITC
Id. sp TCR
18.2%
FSC-A
CD19 PE-cy7
B cells
56.6%
C. B cell subpopulation gating in BM
SSC-A
FSC-A
Lymphocytes
29.9%
FSC-A
FSC-W
Single cells
91%
B220 PerCP
CD19 PE-cy7
B cells
20.5%
IgM APC
CD93 PE
Mature
11.4%
Immature
22.7%
Pre/pro
62.7%
D. B cell subpopulation gating in spleen
(Gated on CD19⁺)
CD93 PE
CD23 PE-cy7
CD21 FITC
IgM APC
T1
5.59%
T3
4.36%
T2
5.32%
MZ
2.51%
B1
1.28%
Fo
71.6%

**Supplementary Figure 1. Gating strategy for definition of cell populations by flow cytometry.**

**A.** Gating of T cell sub populations in thymus (DP: Double positive, CD4SP: CD4 single positive, CD8SP: CD8 single positive. The example is a non-transgenic mouse; see Suppl. Fig. 2A-B and Fig.1C

**B.** Gating of Id-specific T cells, and B cells, in spleen and LN. The example is a Vλ2$^{315m+/-}$Id-sp.TCR-TG$^{+/-}$ mouse, see Suppl. Fig. 2C and Fig. 1C. Id-specific T cells were gated as described here in Fig. 1D-F, 2B, 4C-F, 7F and 7N. B cells were gated as described here in Fig. 2C, 3A-G, 3I, 3M-O.

**C.** Gating of B cell subpopulations in the bone marrow. Pre/pro: CD93$^{+}$ IgM$^{-}$, immature: CD93$^{+}$ IgM$^{+}$, mature: CD93$^{-}$ IgM$^{+}$. The example is Vλ2$^{315m+/-}$ mouse, see Suppl. Fig. 2E. Gating was done as described in[1].

**D.** B cells from spleen were gated as described in **B** and then gated further based on their expression of CD23 and CD93 as described in[1]. From this plot, the cell populations in each quadrant were analyzed for CD21 and IgM expression. FO: follicular B cells, MZ: marginal zone B cells. Subpopulations were defined as follows: T1: CD93$^{+}$ CD23$^{-}$ CD21$^{-}$ IgM$^{+}$; T2: CD93$^{+}$ CD23$^{+}$ CD21$^{lo}$ IgM$^{hi}$, T3: CD93$^{+}$ CD23$^{+}$ CD21$^{lo}$ IgM$^{int}$, FO: CD93$^{-}$ CD23$^{+}$ CD21$^{lo}$ IgM$^{+}$, MZ: CD93$^{-}$ CD23$^{-}$, CD21$^{hi}$ IgM$^{hi}$ and B1: CD93$^{-}$ CD23$^{-}$ CD21$^{int}$ IgM$^{int}$. The example is a Vλ2$^{315m+/-}$ mouse, see Suppl. Fig. 2F.

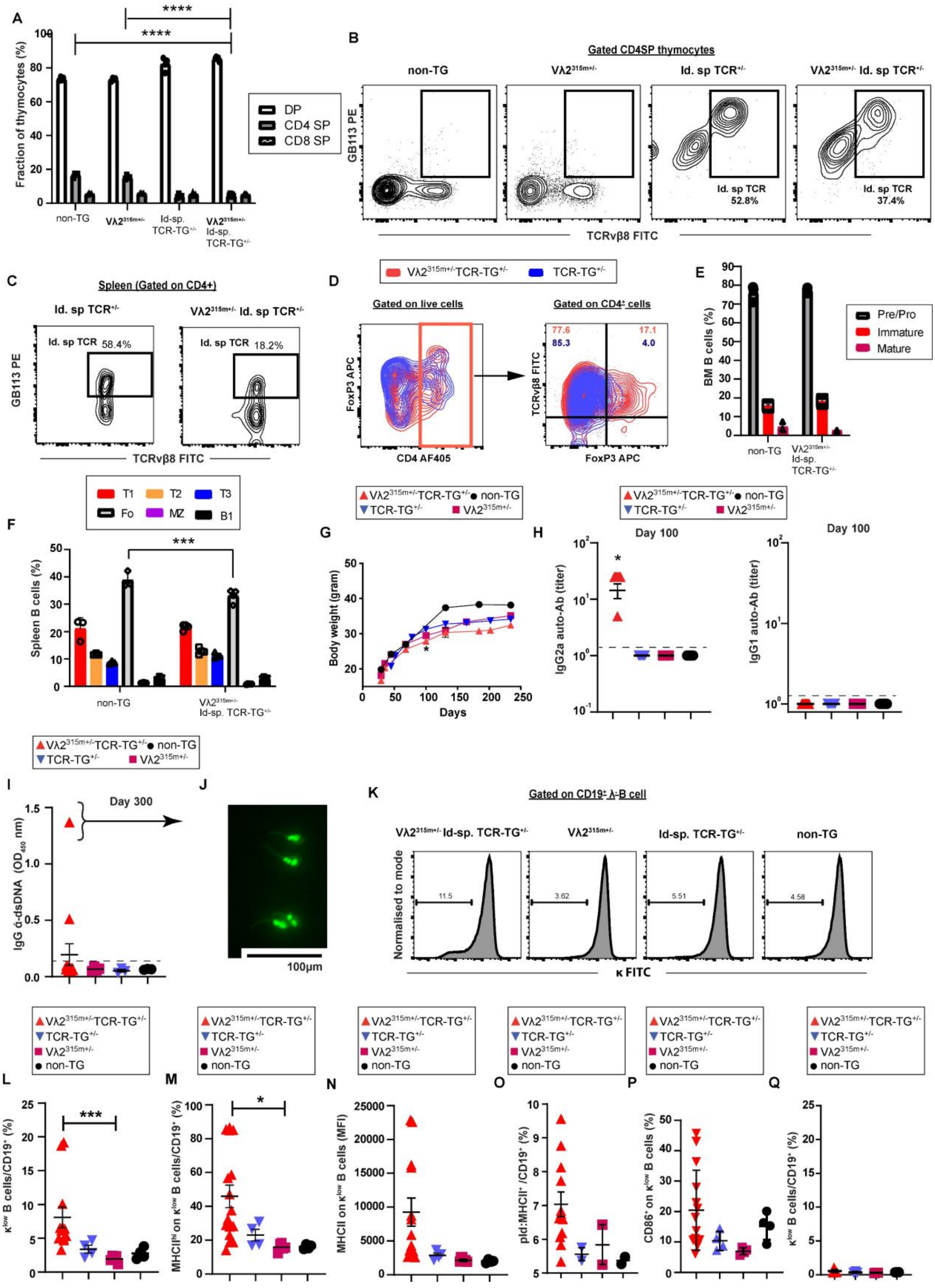
A
Fraction of thymocytes (%)
DP
CD4 SP
CD8 SP
non-TG
Vλ2315m+/-
Id-sp. TCR-TG+/-
Vλ2315m+/- Id-sp. TCR-TG+/-
B
Gated CD4SP thymocytes
non-TG
Vλ2315m+/-
Id. sp TCR+/-
Vλ2315m+/- Id. sp TCR+/-
GB113 PE
Id. sp TCR 52.8%
Id. sp TCR 37.4%
TCRvβ8 FITC
C
Spleen (Gated on CD4+)
Id. sp TCR+/-
Vλ2315m+/- Id. sp TCR+/-
Id. sp TCR 58.4%
Id. sp TCR 18.2%
GB113 PE
TCRvβ8 FITC
D
Vλ2315m+/-TCR-TG+/-
TCR-TG+/-
Gated on live cells
Gated on CD4+ cells
FoxP3 APC
CD4 AF405
TCRvβ8 FITC
77.6
85.3
17.1
4.0
E
BM B cells (%)
Pre/Pro
Immature
Mature
non-TG
Vλ2315m+/- Id-sp. TCR-TG+/-
F
T1
T2
T3
Fo
MZ
B1
Spleen B cells (%)
non-TG
Vλ2315m+/- Id-sp. TCR-TG+/-
G
Vλ2315m+/-TCR-TG+/-
non-TG
TCR-TG+/-
Vλ2315m+/-
Body weight (gram)
Days
H
Day 100
IgG2a auto-Ab (titer)
IgG1 auto-Ab (titer)
I
IgG α-dsDNA (OD450 nm)
Day 300
J
100µm
K
Gated on CD19+ λ-B cell
Vλ2315m+/- Id-sp. TCR-TG+/-
Vλ2315m+/-
Id-sp. TCR-TG+/-
non-TG
Normalised to mode
11.5
3.62
5.51
4.58
κ FITC
L
κlow B cells/CD19+ (%)
M
MHCIIhi on κlow B cells/CD19+ (%)
N
MHCII on κlow B cells (MFI)
O
pId:MHCII+ /CD19+ (%)
P
CD86+ on κlow B cells (%)
Q
κlow B cells/CD19+ (%)

**Supplementary figure 2. Partial deletion of Id-specific CD4$^+$ T cells and increased FoxP3 expression. Autoantibodies and B cell activation.**

**A.** Graph showing T cell sub-populations in thymi of 4-week-old mice. Vλ$^{2315m+/-}$ TCR-TG$^{+/-}$, n=5 and control groups, n=4. p=0.0001 Mean (±SD). **B.** Contour blots showing staining of gated CD4 single positive (SP) thymocytes with GB113 mAb clonotype-specific for the Id-specific transgene-encoded TCR and anti-TCRVβ8 mAb specific for the TCR-transgenic β chain. Representative mice of the indicated four types of mice are shown. See Fig. 1C for comparison of groups of mice and statistics. **C**. The same staining as in B (above) was applied on gated CD4$^+$ spleen T cells. Representative mice are shown. See Fig. 1D for comparison of groups of mice and statistics. **D.** FoxP3 expression among TCRVβ8$^+$ CD4$^+$ T cells in indicated types of 4-week-old mice. See Fig. 1E for comparison of groups of mice and statistics. **E-F.** B cell sub-populations in BM (**E**) and spleen (**F**) of 4-week-old mice (gated as shown in Suppl. Fig. 1C and D). Vλ$^{2315m+/-}$ TCR-TG$^{+/-}$, n=4 and control groups, n=3. Mean (±SD). One representative experiment out of two is shown. F. p=0.0004 **G**. Weight curves of the indicated types of male mice. n=6/group. p<0.0444 (Day 100); Mean (±SEM). **H**. IgG2a$^+$ and IgG1$^+$ autoantibodies in sera of 100 days old mice, detected by staining of HEp-2 cells. See Fig. 1I for day 300 data. Vλ$^{2315m+/-}$ TCR-TG$^{+/-}$, n=9 and control groups, n=4. p=0.0217 (IgG2a). Mean (±SEM). **I**. Detection of anti-dsDNA autoantibodies in sera by ELISA where wells had been coated with dsDNA. Vλ$^{2315m+/-}$ TCR-TG$^{+/-}$, n=15 and control groups, n=6. p=0.2159. Mean (±SEM). **J**. CLIFT assay showing positive staining with serum from one mouse which had anti-dsDNA autoantibodies detected in ELISA (arrow). Scale bar: 100µm. **K, L**. Expansion of κ$^{low}$ B cells (CD19-gated) in peripheral blood of an 11-months-old Vλ2$^{315m+/-}$ TCR-TG$^{+/-}$ mouse (**K**), and statistical comparison between types of mice (**L**). **M-P**. Percentages of MHCII$^{hi}$ (**M,** MFI of MHCII is shown in **N**), pId:MHCII$^+$ (**O**) and CD86$^+$ cells (**P**) among peripheral κ$^{low}$ B cells at 11-month-old mice of the four different types. **L.** Vλ2$^{315m+/-}$ TCR-TG$^{+/-}$, n=12; Vλ2$^{315m+/-}$, n=5; TCR-TG$^{+/-}$, n=4; non-TG, n=4. Mean (±SEM). p=0.0005 and 0.0204. **M.** Vλ2$^{315m+/-}$ TCR-TG$^{+/-}$, n=14; Vλ2$^{315m+/-}$, n=5; TCR-TG$^{+/-}$, n=4; non-TG, n=4. Mean (±SEM). **N.** Vλ2$^{315m+/-}$ TCR-TG$^{+/-}$, n=14; Vλ2$^{315m+/-}$, n=5; TCR-TG$^{+/-}$, n=4; non-TG, n=4. Mean (±SEM). **O.** Vλ2$^{315m+/-}$ TCR-TG$^{+/-}$, n=12; Vλ2$^{315m+/-}$, n=5; TCR-TG$^{+/-}$, n=4; non-TG, n=4. Mean (±SEM). **P.** Vλ2$^{315m+/-}$ TCR-TG$^{+/-}$, n=14; Vλ2$^{315m+/-}$, n=5; TCR-TG$^{+/-}$, n=4; non-TG, n=4. Mean (±SEM). P=0.0434. **Q**. κ$^{low}$ B cells (CD19-gated) in spleens of 2-months-old Vλ2$^{315+/-}$ TCR-TG$^{+/-}$ mice and age-matched controls. Vλ2$^{315m+/-}$ TCR-TG$^{+/-}$, n=4 and control groups n=3. Statistical comparisons: Two-way ANOVA: **C, F**; Kruskal-Wallis

test (two-sided), Dunn's multiple comparisons: **H-I, L-Q**. *$P$<0.05, *** $P$<0.001, **** $P$<0.0001. Source data are provided as a Source Data file.

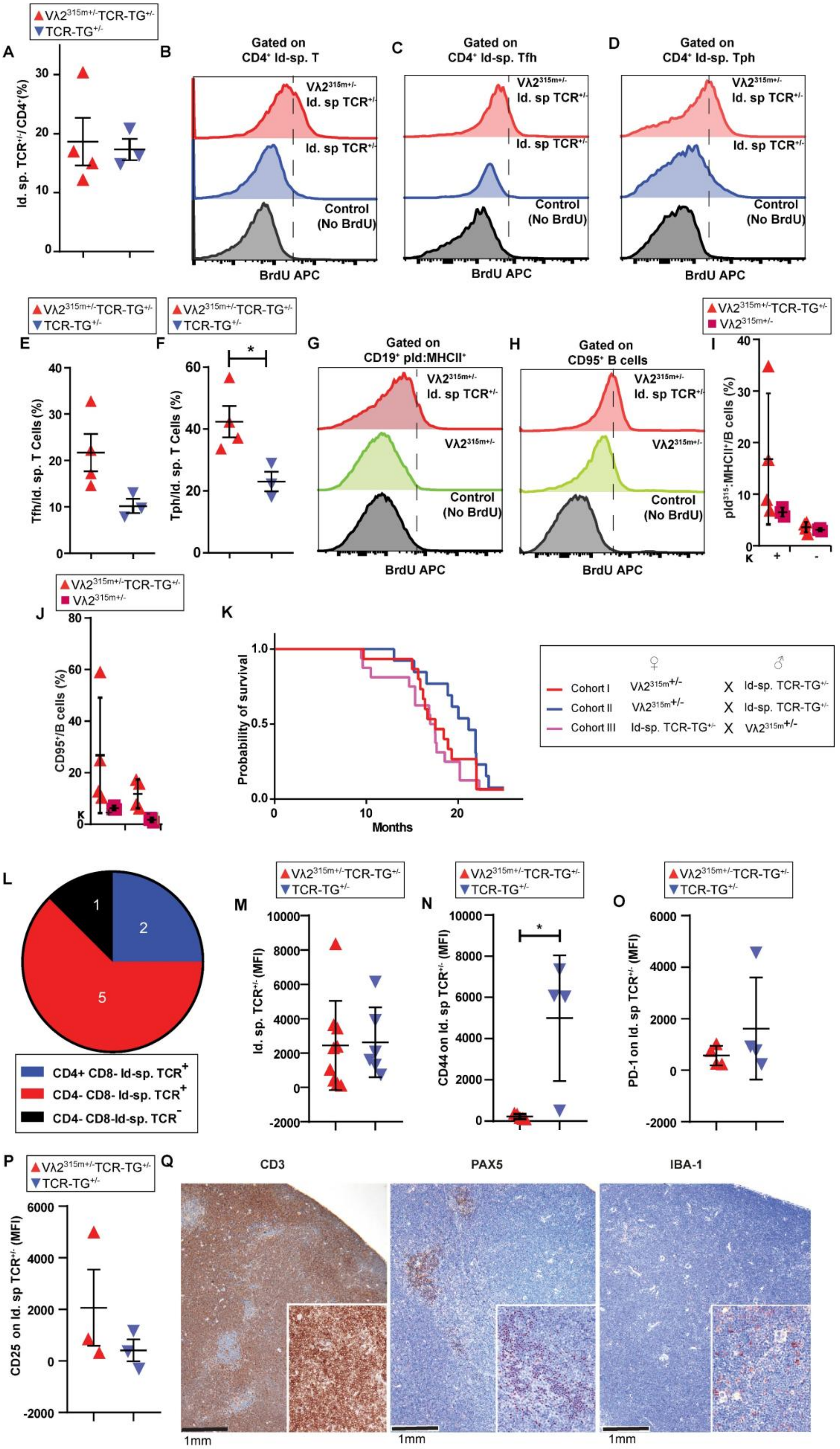

A
B
C
D
E
F
G
H
I
J
K
L
M
N
O
P
Q
Gated on CD4+ Id-sp. T
Gated on CD4+ Id-sp. Tfh
Gated on CD4+ Id-sp. Tph
Gated on CD19+ pId:MHCII+
Gated on CD95+ B cells
BrdU APC
Control (No BrdU)
Probability of survival
Months
Cohort I
Cohort II
Cohort III
CD4+ CD8- Id-sp. TCR+
CD4- CD8- Id-sp. TCR+
CD4- CD8-Id-sp. TCR-
CD3
PAX5
IBA-1
1mm

**Supplementary Figure 3. Proliferation of T and B cell subsets in pre-lymphoma mice. Characterization of T lymphomas.**

**A.** Frequencies of Id-specific $CD4^+$ in spleens from 12-months-old $V\lambda2^{315m+/-}$ TCR-$TG^{+/-}$ and TCR-$TG^{+/-}$ mice. $V\lambda2^{315m+/-}$ TCR-$TG^{+/-}$, n=4 and TCR-$TG^{+/-}$, n=3. Mean (±SEM). **B**. BrdU incorporation into splenic Id-specific $CD4^+$ $GB113^+$ (clonotypic mAb) cells in 12-months-old $V\lambda2^{315m+/-}$ TCR-$TG^{+/-}$ and TCR-$TG^{+/-}$ mice at day 7 after a BrdU pulse. Representative examples are shown. See Fig. 2A for comparison of groups of mice and statistics. **C.** BrdU incorporation into splenic $CD4^+$ Id-specific Tfh cells ($CXCR5^+$PD-$1^+$) in $V\lambda2^{315m+/-}$ TCR-$TG^{+/-}$ and TCR-$TG^{+/-}$ mice. Representative examples are shown. See Fig. 2B for comparison of groups of mice and statistics. **D.** BrdU incorporation into splenic $CD4^+$ Id-specific Tph cells ($CXCR5^-$PD-$1^+$) in $V\lambda2^{315m+/-}$ TCR-$TG^{+/-}$ and TCR-$TG^{+/-}$ mice. Right: Representative examples are shown. Left: Frequency of $BrdU^+$ cells among Tph. n=3-4/group. **E-F.** Frequencies of Tfh (**E**) and Tph (**F**) among splenic Id-specific $CD4^+$ cells. $V\lambda2^{315m+/-}$ TCR-$TG^{+/-}$, n=4 and TCR-$TG^{+/-}$, n=3. p=0.0668 (**E**) and 0.0316 (**F**). Mean (±SEM). **G**. BrdU incorporation into $pId^{315}$:$MHCII^+$ B cells in $V\lambda2^{315m+/-}$ TCR-$TG^{+/-}$ and $V\lambda2^{315m+/-}$ mice. $pId^{315}$:MHCII was detected by a TCRm reagent. Representative examples are shown. See Fig. 2C for comparison of groups of mice and statistics. **H**. BrdU incorporation into $CD95^+$ B cells in $V\lambda2^{315m+/-}$ TCR-$TG^{+/-}$ and $V\lambda2^{315m+/-}$ mice. Right: Representative examples are shown.Left: Frequency of $BrdU^+$ cells among $CD95^+$ B cells. $V\lambda2^{315m+/-}$ TCR-$TG^{+/-}$, n=4 and $V\lambda2^{315m+/-}$, n=3. **I-J.** Frequencies of $pId^{315}$:$MHCII^+$ (**I**) and $CD95^+$ (**J**) B cells among $CD19^+$ spleen cells. $\kappa^+$ and $\kappa^-$ cells were analyzed. $V\lambda2^{315m+/-}$ TCR-$TG^{+/-}$, n=4 and $V\lambda2^{315m+/-}$, n=3.. Mean (±SD). **K**. Survival graphs of $V\lambda2^{315m+/-}$ Id-sp. TCR-$TG^{+/-}$ mice from three individual cohorts. For each of the cohorts, the sex of the parent is indicated (box). cohort I, n=15; cohort II, n=13 and cohort III, n=16. **L-P**. Characterization of T lymphomas. **L.** Classification of T lymphomas based on their TCR, CD4 and CD8 expression. n=8/group. **M-P.** Expression of different activation markers on gated Id-sp. TCR-$TG^+$ cells in T lymphomas. MFI of Id-sp. $TCR^+$ (**M**), CD44 (**N**), PD-1 (**O**) and CD25 (**P**) are shown. **M.** $V\lambda2^{315m+/-}$ TCR-$TG^{+/-}$, n=6; TCR-$TG^{+/-}$, n=9. **N.** $V\lambda2^{315m+/-}$ TCR-$TG^{+/-}$, n=4; TCR-$TG^{+/-}$, n=5. p=0.0498 (**N**). **O.** n=4/group. **P.** n=3/group. Mean (±SEM). **Q**. Immunohistochemistry staining of T-LBL for CD3, PAX5 and IBA-1. The T-LBL stains extensively for the T cell marker CD3 while normal $PAX5^+$ B cells and IBA-1 histiocytes are scarce. Scale bar: 1mm. Statistical comparisons: Mann-Whitney U test (two-tailed): **A,C-F,H-J,M-P** . *$P$<0.05. Source data are provided as a Source Data file.

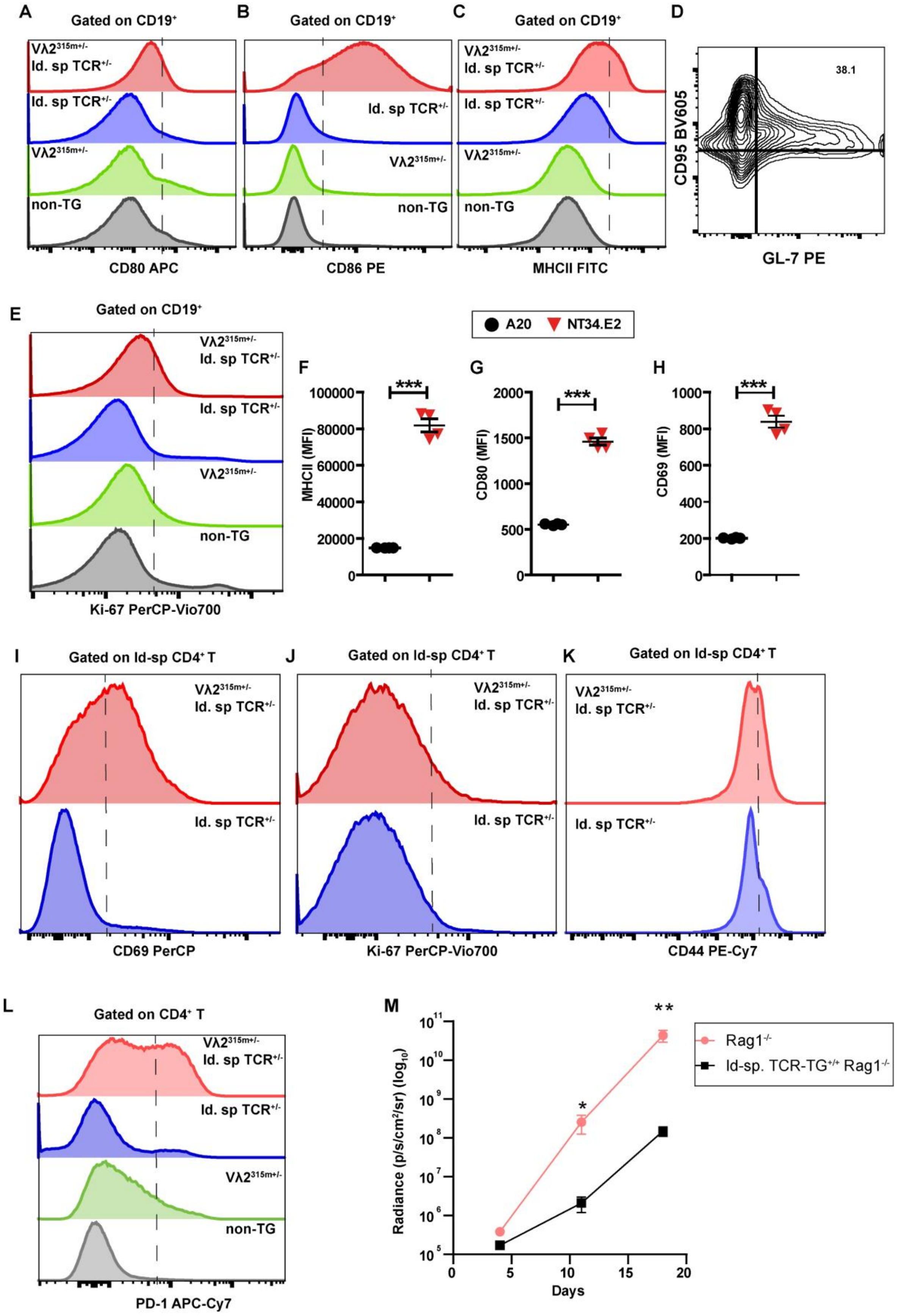


**Supplementary figure 4. Markers on B lymphomas and infiltrating T cells. Tumor challenge experiment.**

**A-E.** Stainings of primary B cell lymphomas in $V\lambda 2^{315m+/-}$ TCR-$TG^{+/-}$ mice. **(A-C).** Representative histograms showing expression of CD80 (**A**), CD86 (**B**) and MHCII (**C**) in CD19-gated splenic B cells of the four types of mice. **D**. Representative contour plot showing expression of GC markers CD95 and GL-7 in a $V\lambda 2^{315m+/-}$ TCR-$TG^{+/-}$ mouse with primary splenic B lymphoma. **E.** Representative histograms showing expression of Ki-67 in the four types of mice. See Fig. 3E for groups of mice and statistical analysis. **F-H.** Expression of MHCII (**F**), CD80 (**G**) and CD69 (**H**) on cloned B lymphoma cells NT34.E2 derived *in vitro* from the spleen of a $Rag1^{-/-}$ mouse transferred with primary B lymphoma cells. A20 B lymphoma cells served as a control. n=4/group, technical duplicates. One representative experiment out of three experiments. **F-H.** $p<0.0001$. Mean (±SD). **I-K.** Characterization of tumor infiltrating T cells in primary B cell lymphomas. Representative histograms showing expression of CD69 (**I**), Ki-67 (**J**), CD44 (**K**) and PD-1 (**L**) in gated Id-sp. $CD4^{+}$ T cells in $V\lambda 2^{315m+/-}$ TCR-$TG^{+/-}$ compared to control mice. See Fig. 4C-F for groups and statistics. **M.** Graph showing the tumor bioluminescence radiance in $Rag1^{-/-}$ mice compared to Id-sp. TCR-$TG^{+/+}Rag1^{-/-}$ mice injected i.v. with NT34 cells labeled with luciferase. n=4/group. p=0.0286 (day 14) and 0.0014 (day 18). Mean (±SD). Statistical comparisons: Unpaired student T test (two-tailed): **F-H**, Two-way ANOVA: **M**. *$P<0.05$, ** $p<0.01$, *** $p<0.001$, **** $p<0.0001$. Source data are provided as a Source Data file.

**A. NT7**

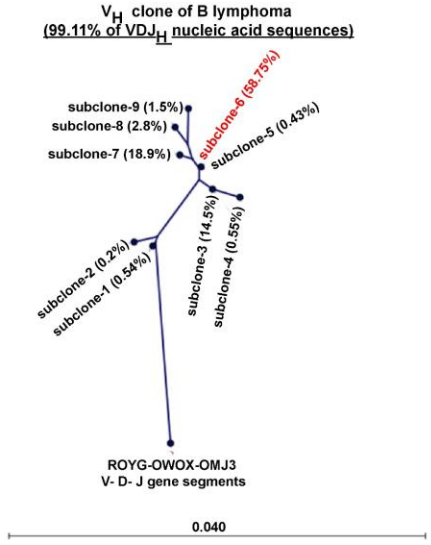

V_H clone of B lymphoma
(99.11% of VDJ_H nucleic acid sequences)
subclone-9 (1.5%)
subclone-8 (2.8%)
subclone-7 (18.9%)
subclone-6 (58.75%)
subclone-5 (0.43%)
subclone-3 (14.5%)
subclone-4 (0.55%)
subclone-2 (0.2%)
subclone-1 (0.54%)
ROYG-OWOX-OMJ3
V- D- J gene segments
0.040


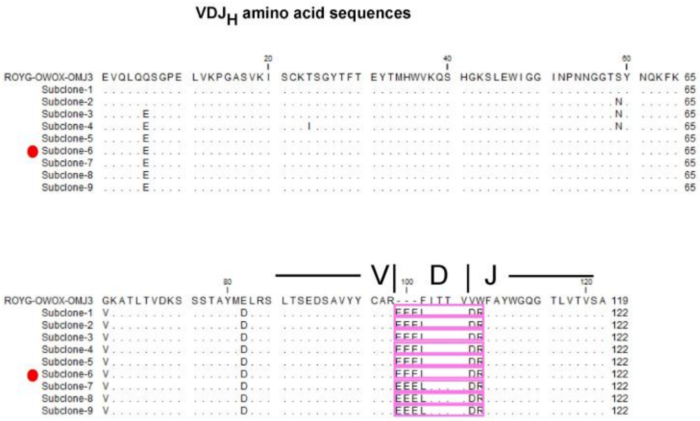

VDJ_H amino acid sequences
20
40
60
ROYG-OWOX-OMJ3 EVQLQQSGPE LVKPGASVKI SCKTSGYTFT EYTMHWVKQS HGKSLEWIGG INPNNGGTSY NQKFK 65
Subclone-1 65
Subclone-2 N 65
Subclone-3 E N 65
Subclone-4 E I N 65
Subclone-5 E 65
Subclone-6 E 65
Subclone-7 E 65
Subclone-8 E 65
Subclone-9 E 65
V D J
80
100
120
ROYG-OWOX-OMJ3 GKATLTVDKS SSTAYMELRS LTSEDSAVYY CAR---FITT VVWFAYWGQG TLVTVSA 119
Subclone-1 V D EEEL DR 122
Subclone-2 V D EEEL DR 122
Subclone-3 V D EEEL DR 122
Subclone-4 V D EEEL DR 122
Subclone-5 V D EEEL DR 122
Subclone-6 V D EEEL DR 122
Subclone-7 V D EEEL DR 122
Subclone-8 V D EEEL DR 122
Subclone-9 V D EEEL DR 122


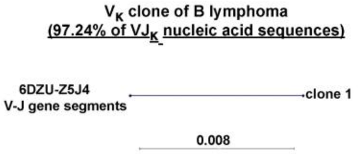

V_K clone of B lymphoma
(97.24% of VJ_K nucleic acid sequences)
6DZU-Z5J4
V-J gene segments
clone 1
0.008


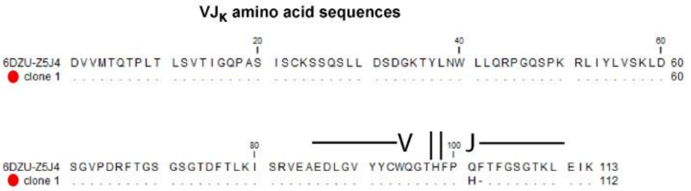

VJ_K amino acid sequences
20
40
60
6DZU-Z5J4 DVVMTQTPLT LSVTIGQPAS ISCKSSQSLL DSDGKTYLNW LLQRPGQSPK RLIYLVSKLD 60
clone 1 60
V J
80
100
6DZU-Z5J4 SGVPDRFTGS GSGTDFTLKI SRVEAEDLGV YYCWQGTHFP QFTFGSGTKL EIK 113
clone 1 H- 112

**B. NT8**

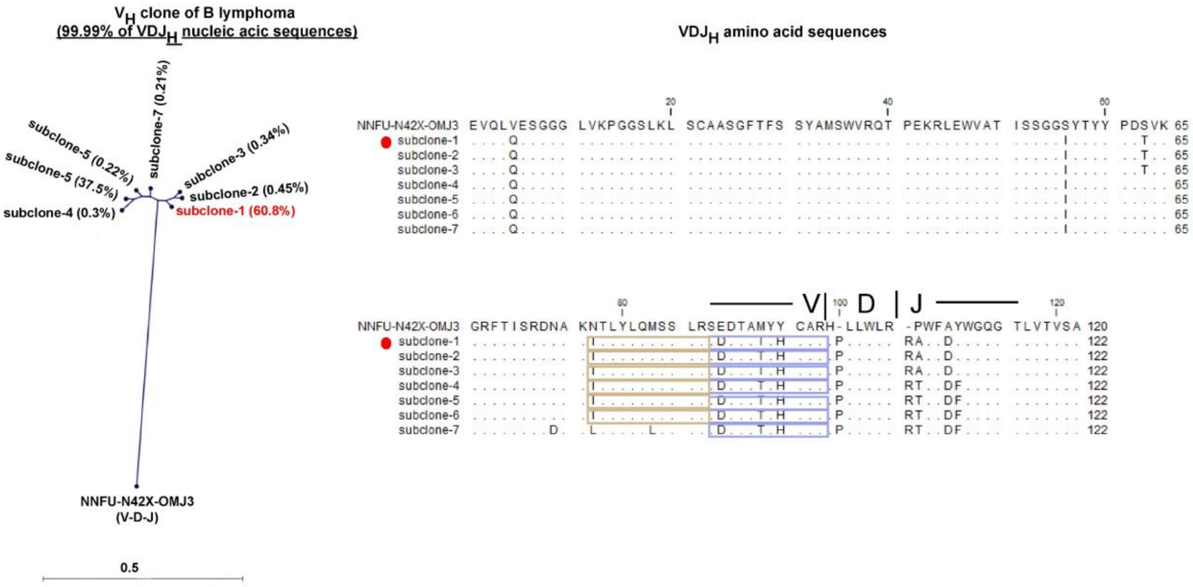

$V_H$ clone of B lymphoma
(99.99% of $VDJ_H$ nucleic acic sequences)
subclone-7 (0.21%)
subclone-5 (0.22%)
subclone-5 (37.5%)
subclone-3 (0.34%)
subclone-2 (0.45%)
subclone-4 (0.3%)
subclone-1 (60.8%)
NNFU-N42X-OMJ3
(V-D-J)
0.5
$VDJ_H$ amino acid sequences
NNFU-N42X-OMJ3 EVQLVESGGG LVKPGGSLKL SCAASGFTFS SYAMSWVRQT PEKRLEWVAT ISSGGSYTYY PDSVK 65
V D J
NNFU-N42X-OMJ3 GRFTISRDNA KNTLYLQMSS LRSEDTAMYY CARH-LLWLR -PWFAYWGQG TLVTVSA 120


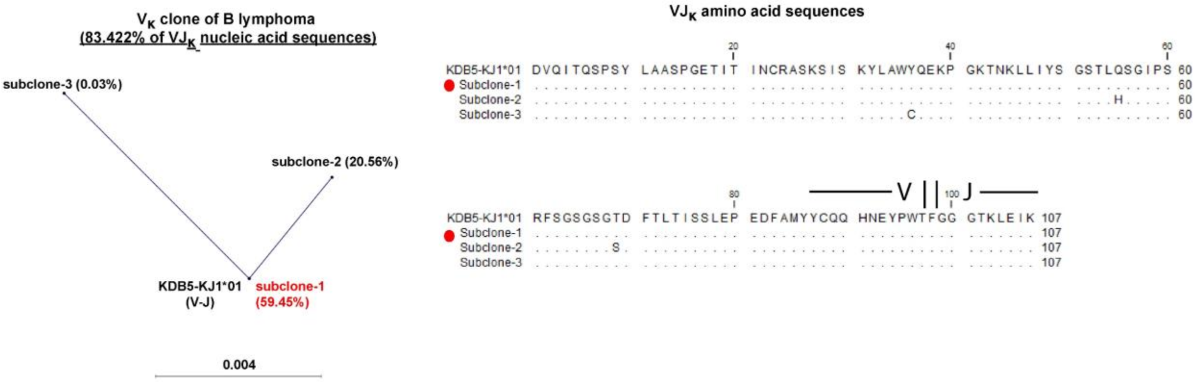

$V_K$ clone of B lymphoma
(83.422% of $VJ_K$ nucleic acid sequences)
subclone-3 (0.03%)
subclone-2 (20.56%)
KDB5-KJ1*01
(V-J)
subclone-1
(59.45%)
0.004
$VJ_K$ amino acid sequences
KDB5-KJ1*01 DVQITQSPSY LAASPGETIT INCRASKSIS KYLAWYQEKP GKTNKLLIYS GSTLQSGIPS 60
V J
KDB5-KJ1*01 RFSGSGSGTD FTLTISSLEP EDFAMYYCQQ HNEYPWTFGG GTKLEIK 107

**C. NT18**

$V_H$ clone of B lymphoma
(75.09% of $VDJ_H$ nucleic acid sequences)

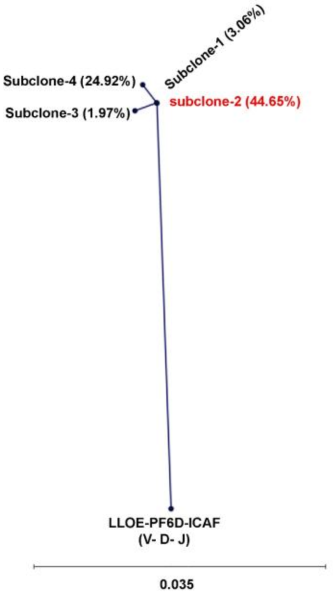


$VDJ_H$ amino acid sequences

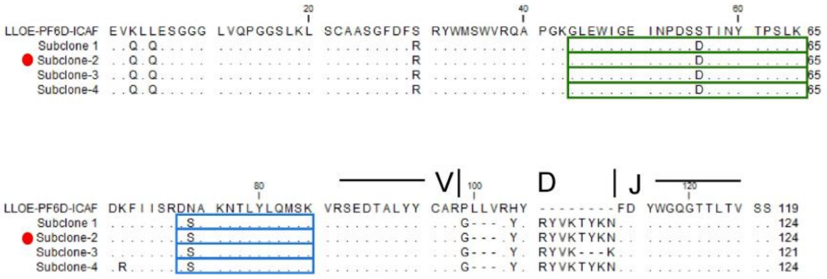


$V_K$ clone of B lymphoma
(90.05% of $VJ_K$ nucleic acid sequences)

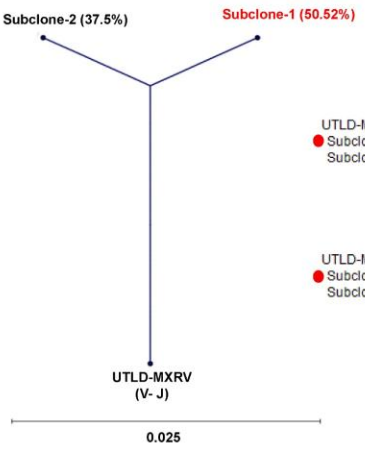


$VJ_K$ amino acid sequences

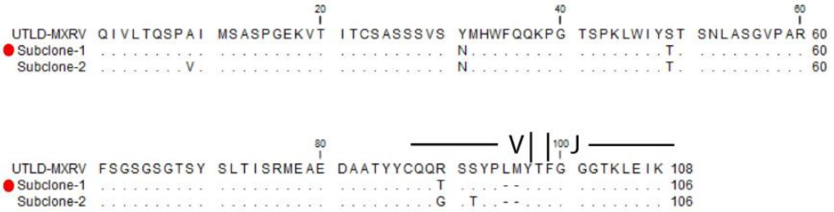

D. NT24

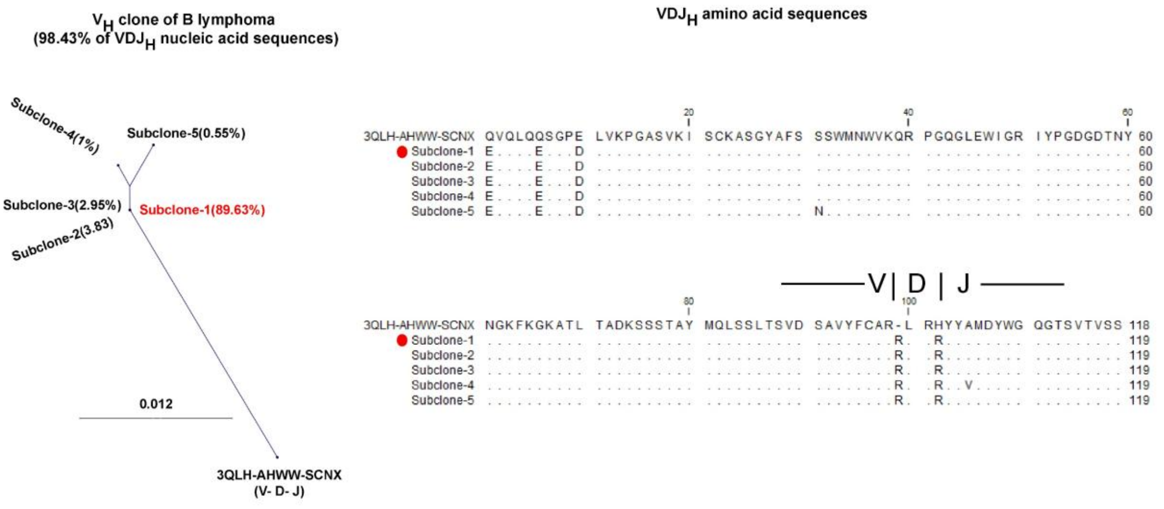


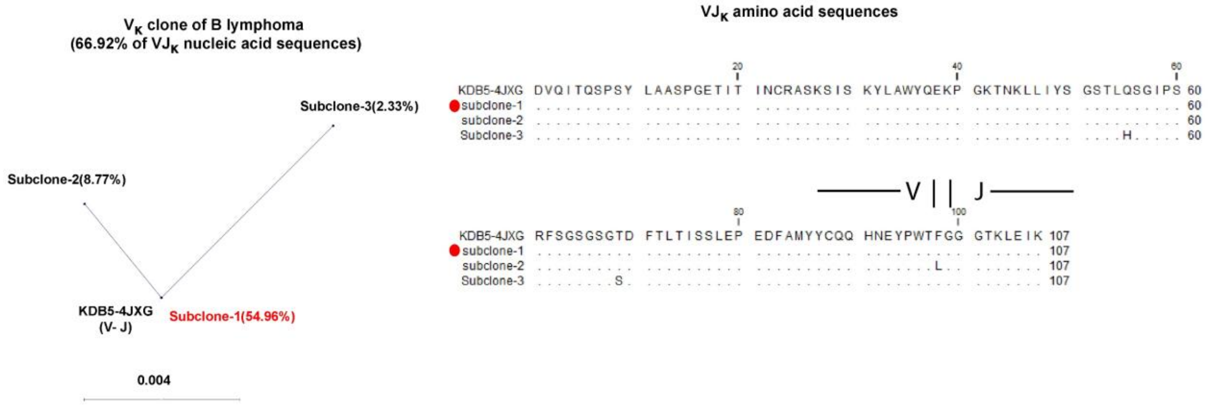


**Supplementary Figure 5. Dominant VDJ$_H$ and VJ$_k$ sequences of B lymphomas.** Primary B lymphomas were injected i.v. into Rag1$^{-/-}$ mice. The lymphoma B cell clones were propagated *in vivo*. Once the mice reached the humane end point, singles cells were prepared from the spleens. RNA was isolated and VDJ$_H$ and V$_\kappa$J$_\kappa$ sequences were amplified using specific primers and sequenced. The nucleic acid sequences have been submitted to Genbank (accession numbers: PV068692 - PV068753 and PV068754 - PV068825). **A**. NT7 B lymphoma. A total of 25976 reads for VDJ$_H$ and 31018 reads for VJ$_k$ nucleotide sequences were analyzed. Top left: a radial tree showing the clonal evolution of VDJ$_H$ nucleotide sequences, starting with the closest BALB/c germline VDJ$_H$ (ROYG-OWX-OMJ3)[2] . Only subclones with more than 50 reads were included in the analysis. Frequencies are indicated in parentheses. The most frequent (dominant) subclone is indicated in red. Top right: Translated

amino acid sequences of all the subclones have been listed according to their position in the radial tree. Bottom left: a radial tree showing the clonal evolution of $VJ_k$ nucleotide sequences from the closest BALB/c germline $VJ_k$ (6DZU-Z5J4) [2]. Only subclones with more than 50 reads were included in the analysis. Frequencies are indicated in parentheses. The most frequent (dominant) subclone is indicated in red. Bottom right: Translated amino acid sequences of all the subclones have been listed according to their position in the radial tree. The most frequent (dominant) subclone is indicated in red. **B**. NT8 B lymphoma. A total of 38441 reads for $VDJ_H$ and 2057 reads for $VJ_k$ reads were analyzed. Data are presented as in **A**., above. NT8 had another $VJ_k$ (12.5%) composed of the same V gene segment (KDB5) but a different Jκ (Z5J4, MXRV), this $VJ_k$ is not shown. **C**. NT18 B lymphoma. A total of 40428 reads for $VDJ_H$ and 70229 and $VJ_k$ reads were analyzed. Data are presented as in **A**., above. **D**. NT24 B lymphoma. A total of 36594 1reads for $VDJ_H$ and 2485 and $VJ_k$ reads were analyzed. Data are presented as in **A**., above. NT24 had another $VJ_k$ (31.38%) composed of the same V gene segment (KDB5) but a different Jκ (MXRV) this $VJ_k$ is not shown..

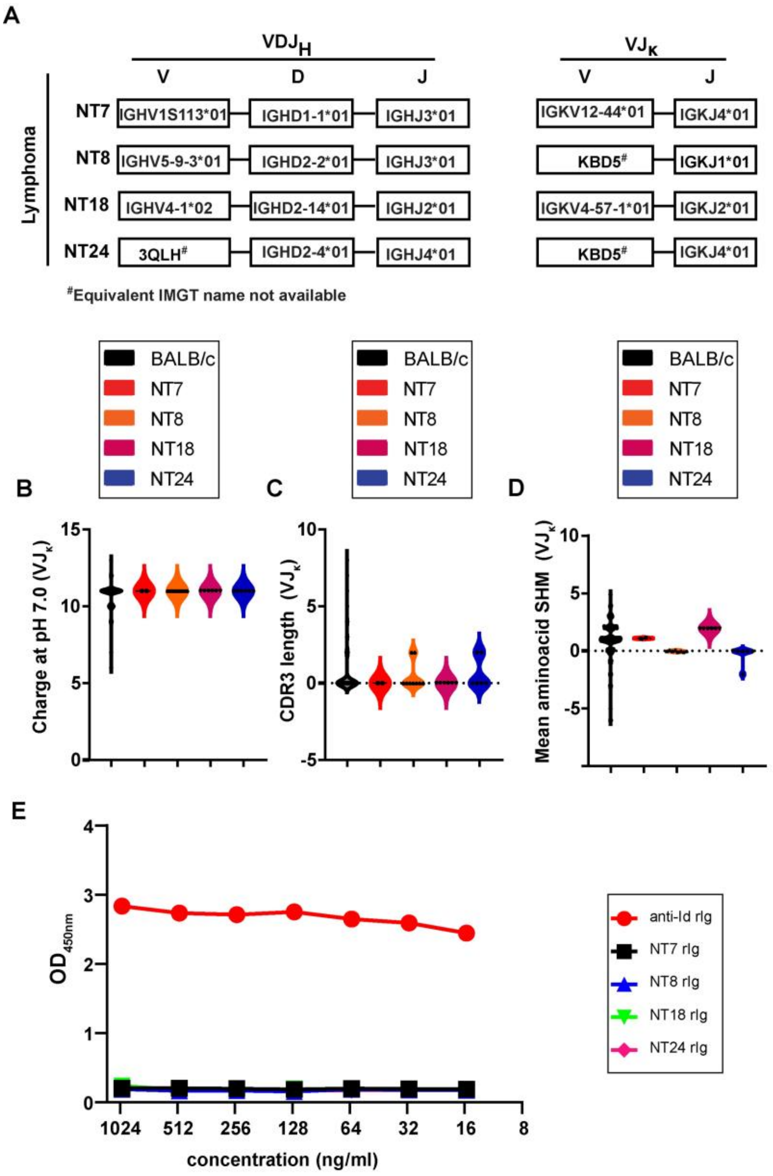


**Supplementary Figure 6. Further characterization of lymphoma BCR.**

**A.** IMGT names for the $V_H$, $D_H$, $J_H$ and $V_k$, $J_k$ gene segments used by the NT7, NT8, NT18 and NT24 B lymphomas. **B-D**. Characterization of dominant $VJ_k$ of the four indicated lymphomas: net charge at pH 7.0 **(B)**, CDR3 length **(C)** and expressed SHM (**D**). BALB/c, n=380; NT3, n=2; NT8, n=9; and NT18 and NT24, n=6. Violin plots show the distribution of individual values. **E.** ELISA plates were coated with $\lambda 2^{315}$ Ig (M315) and four different lymphoma rIgs were added in dilutions. Anti-Id rIg was used as control. The bound rIgs were detected with anti-human κ antibody. Samples were run in duplicates. Mean values are shown. Source data are provided as a Source Data file.

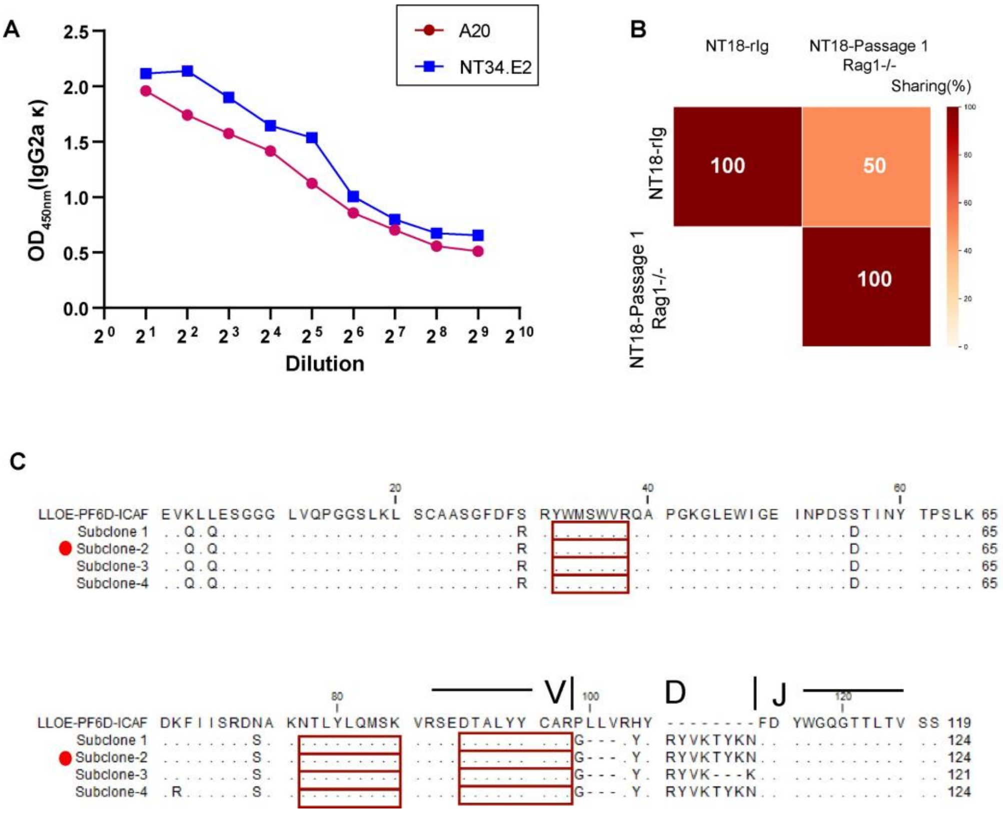


**Supplementary Figure 7. BCR V region-derived peptides are detected in sera of lymphoma mice.**

**A.** The levels of IgG2aκ secreted by *in vitro*-cultured A20 and NT34.E2 B lymphoma cell lines into supernatants were measured by ELISA. Dilution curves from one out of two experiments shown. **B**. Heat map showing the peptide similarity identified by mass spectrometry between NT18 lymphoma rIg and serum of Rag1$^{-/-}$ mice injected *i.v.* with NT18 lymphoma cells and that developed a lymphoma. **C**. Mapping of shared peptides in B (above) to NT18 VDJ$_H$ subclone sequences, indicated by boxes. Subclone 2 (red dot) is the dominant subclone. Source data are provided as a Source Data file.

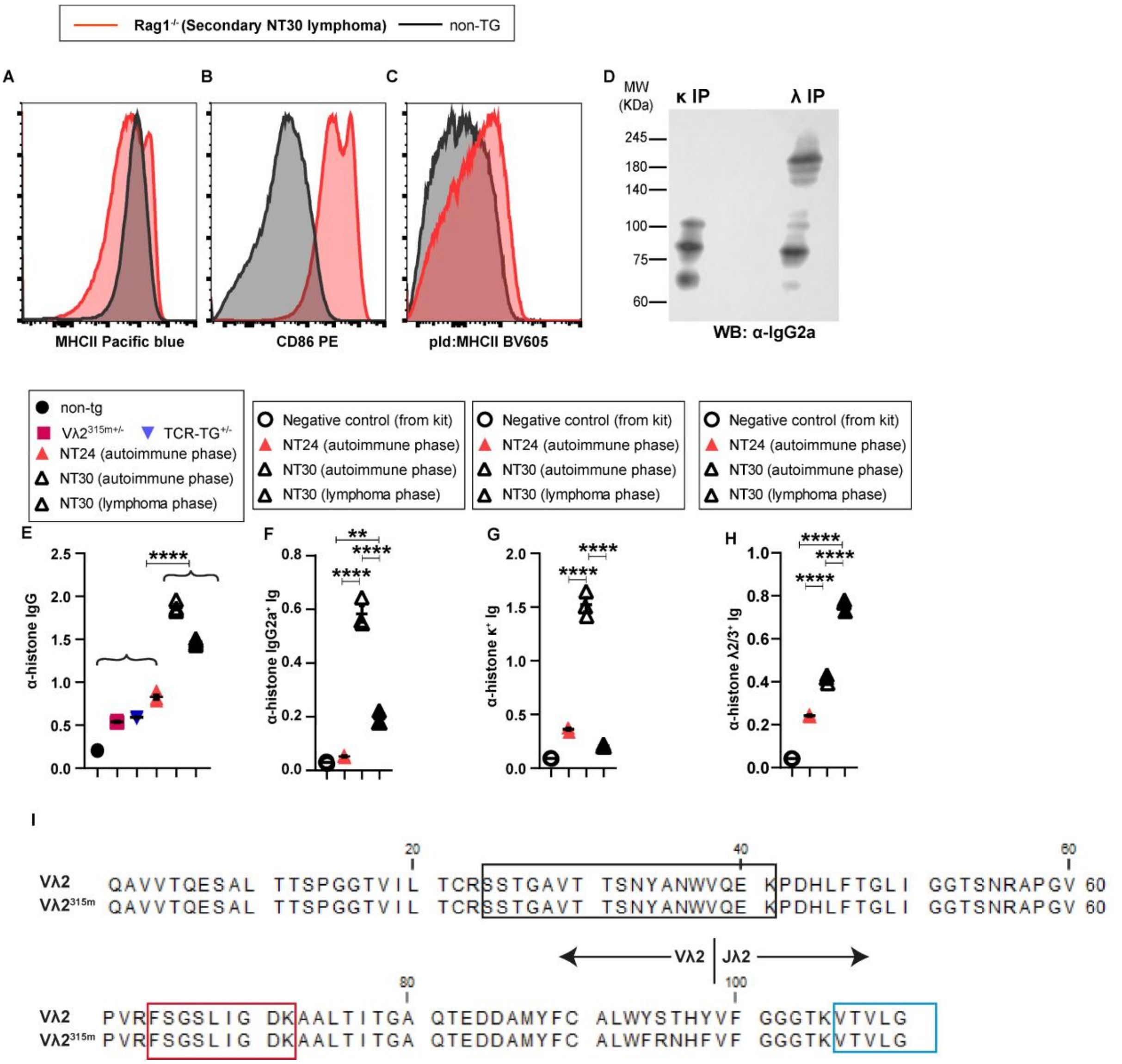


**Supplementary Figure 8. Further characterization of secondary NT30 B cell lymphoma and autoimmune serum from primary NT30 mouse. A-D.** Spleen cells from primary NT30 lymphoma mice were transferred *i.v.* into Rag $1^{-/-}$ mice resulting in a secondary NT30 lymphoma. Expression of MHCII (**A**), CD86 (**B**) and pId:MHCII (**C**) on $CD19^+$ spleen cells of $Rag1^{-/-}$ mice with secondary NT30 lymphoma. **D.** NT30 cell lysate was immunoprecipitated with anti-κ and anti-λ mAbs. Western blot under non-reducing conditions revealed that the IgG2a 180kDa band (probably BCR) was associated with λ but not κ. **E-H.** Histone-specific IgG **(E)**, IgG2a **(F)**, κ **(G)** and λ2/3 **(H)** autoantibodies in autoimmune- and lymphoma-phase sera from the NT30 mouse compared to controls. The control NT24 autoimmune phase serum contains predominantly $\kappa^+$ anti-histone autoAb (NT24 is a $\kappa^+$BCR B lymphoma). Negative control was supplied with the kit. n=3, technical replicates. One of two independent experiments is shown. $p<0.0001$ (**E, G, H**) and $p=0.0019$ and $<0.0001$ (**F**).

Mean (±SD). **I.** Lymphoma phase NT30 serum (death bleed) was precipitated with anti-Vλ (9A8) mAb and digested with trypsin and AspN. Peptides identified by mass spectrometry are boxed and mapped to germline Vλ2, $V\lambda2^{315m}$ and Jλ2 sequences respectively. No Jλ1 peptide was found. This indicates that the L chain of autoantibodies is λ2 and most probably $\lambda2^{315m}$. Statistical comparisons: One-way ANOVA test. ** $p<0.01$, **** $p<0.0001$. Source data are provided as a Source Data file.

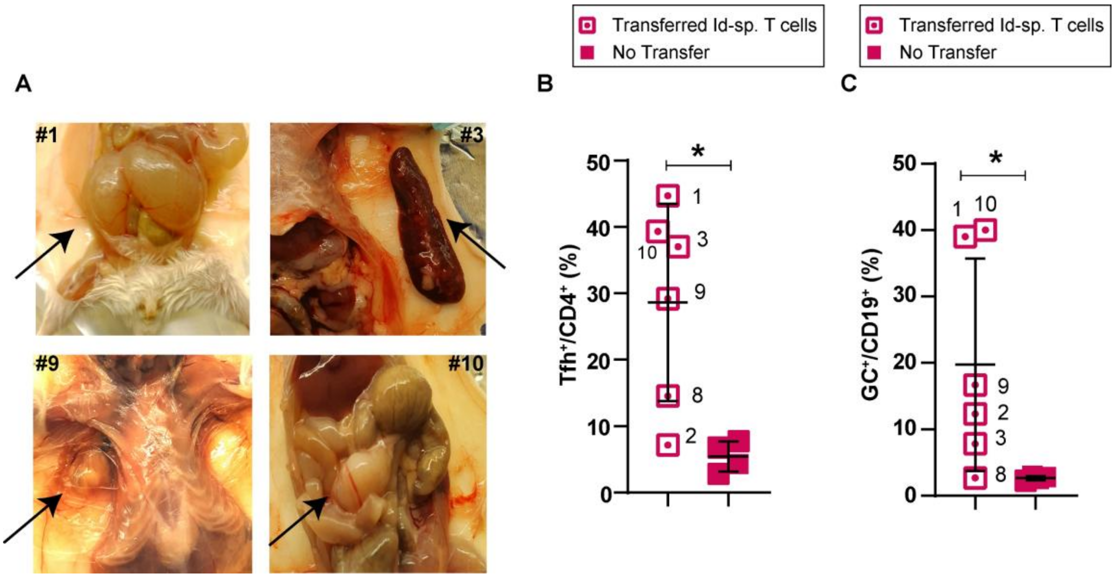


**Supplementary Figure 9. Lymphoma development in Vλ2$^{315m+/-}$ mice transferred with Id-specific CD4$^{+}$ T cells.** (See also Fig. 7N-Q and Supplementary Table 1). **A.** Pictures showing enlarged lymphoid and non-lymphoid organs in the four mice that developed lymphoma. #1: Lower abdominal cyst. #3: Enlarged spleen. #9: Enlarged axillary lymph node. #10: Enlarged Peyer's patches. **B-C**. Analysis of T and B cells in Vλ2$^{315m+/-}$ mice (n=6) injected with Id-specific CD4$^{+}$ T cells compared to non-injected control mice (n=4). For mice that developed B lymphomas, results from the affected lymphoid or non-lymphoid organs are shown, otherwise, analysis is from the spleen (individual T cell transfer mice have been numbered, see Supplementary Table 1). **B.** Tfh CD4$^{+}$ T cells (PD-1$^{+}$ CXCR5$^{+}$). p=0.0190. Mean (±SD). **C.** GC B cells (CD95$^{+}$ GL-7$^{+}$). p=0.0381. Mean (±SD). Mann-Whitney U test (two-tailed), * $p<0.05$. Mean (±SD) are indicated. Source data are provided as a Source Data file.

**Supplementary Table 1. Summary of V$\lambda$2$^{315m+/-}$ mice transferred (or not) with Id-specific CD4 T**[1]

| | | Mouse | 13 months after transfer (autoimmune phase) | | 16-20 months after transfer (lymphoma development) | | | | | | |
|---|---|---|---|---|---|---|---|---|---|---|---|
| | | # | Id-sp. $CD4^+$ in blood (%) | Auto-anti-bodies in serum | Months after transfer | En-larged organs[2] | Id-sp. $CD4^+$ T /$CD4^+$ (%) | $CD19^+$ cells (%) | pId:$MHCII^+$ / $CD19^+$ cells (%) | IHC classification[3] | Conclusion |
| Transfer Id-sp. $CD4^+$ T | Lymphoma | 1 | 12.1 | ND[4] | 19[5] | S | 0.8 | 49.2 | 3.6 | S: DLBCL | DLBCL |
| | | | | | | mLN | 0.8 | 34.4 | 6.3 | | |
| | | | | | | LAC | 28.9 | 4.4 | 27.5 | | |
| | | 3 | 18.5 | ND | 20[6] | S | 0.39 | 49.6 | 11.8 | **S:FBL** | FBL |
| | | 9 | 39.7 | $ANA^+$ | 20[5] | S | 0.6 | 59.1 | 10.9 | S:FBL | Autoimmunity, FBL |
| | | | | | | aLN | 10.9 | 25.3 | 46.7 | | |
| | | 10 | 2.26 | $ANA^+$ | 20[5] | S | 0.8 | 6.6 | 30.7 | P:FBL | Autoimmunity, FBL |
| | | | | | | PP | 8.8 | 37.5 | 80.8 | | |
| | No lymphoma(?) | 2 | NA[7] | NA | 16[5] | S | 0.8 | 30.9 | 7.15 | NA | No lymphoma (?) |
| | | | | | | mLN | 42.1 | 26.7 | 73.1 | | |
| | | 8 | 3.5 | ND | 20[6] | ND | S: 0.3 | 46.9 | 11.4 | ND | No lymphoma (?) |
| No transfer | No Lymphoma | 4 | ND | ND | 16[8] | ND | ND | S: 47.4 | 3.65 | NA | No lymphoma |
| | | | | | | | | LN: 24.9 | 7.3 | | |
| | | 5 | ND | ND | 19[8] | ND | ND | S: 55.5 | 6.5 | NA | No lymphoma |
| | | | | | | | | LN: 25.3 | 7.9 | | |
| | | 6 | ND | ND | 20[8] | ND | ND | S: 40.1 | 6.5 | NA | No lymphoma |
| | | | | | | | | LN: 39.5 | 6.2 | | |
| | | 7 | ND | ND | 20[8] | ND | ND | S: 41 | 3.12 | NA | No lymphoma |
| | | | | | | | | LN: 31.5 | 7.1 | | |

[1]Six two-months-old V$\lambda$2$^{315m+/-}$ mice were transferred *i.v.* with 2x10$^6$ $CD4^+$ spleen T cells from TCR-TG mice amounting to 1x10$^6$ Id-specific $CD4^+$ T cells. Four V$\lambda$2$^{315m+/-}$ mice were not transferred and served as controls. Thirteen months later, mice were investigated for presence of Id-specific $CD4^+$ T cells in blood, and autoantibodies in serum. Starting from 16 months after transfer, some mice became sick, were euthanized, and organs were analyzed by immunohistochemistry (IHC) and flow cytometry.

[2]S: Spleen, mLN: Mesenteric LN, LAC: Lower abdominal cyst, aLN: axillary LN, PP: Peyer's patches. Enlarged organs are pictured in Suppl. Fig. 9A.

[3]DLBCL: Diffuse Large B cell lymphoma, FBL: Follicular B lymphoma.

[4]ND: not detected.

[5] Mice were euthanized upon reaching the endpoint.

[6]Mice were euthanized due to the experimental time limit (20 months after transfer).

[7]NA: not analyzed

[8]Control mice were included in flow cytometry and IHC in each of the three experiments on T cell transferred mice performed 16, 19 and 20 months after transfer.

## References


1 Jacobsen, J. *et al.* Naive idiotope-specific B and T cells collaborate efficiently in the absence of dendritic cells. *J Immunol* **192**, 4174–4183 (2014). https://doi.org/10.4049/jimmunol.1302359

2 Lees, W. IG receptor germline set for species: Mouse subgroup: BALB/c/ByJ set_name: BALB/c/ByJ IGLV (Version 2) [Data set]. 2 ed. (Zenodo, 2024). https://zenodo.org/records/11002140
