# Supplementary material for: Idiotype-specific CD4+ T cells chronically stimulate autoreactive B cells to develop into B lymphomas in mice
Source: Nat Commun. 2026 Feb 25;17:3200. doi: 10.1038/s41467-026-69916-w (PMC13057116; doi:10.1038/s41467-026-69916-w)
Supplement: Supplementary file 3 — Reporting Summary [file 41467_2026_69916_MOESM3_ESM.pdf]

nature portfolio

Corresponding author(s): Ramakrishna Prabhu Gopalakrishnan

Last updated by author(s): 08/01/2026

Reporting Summary

Nature Portfolio wishes to improve the reproducibility of the work that we publish. This form provides structure for consistency and transparency in reporting. For further information on Nature Portfolio policies, see our Editorial Policies and the Editorial Policy Checklist.

Statistics

For all statistical analyses, confirm that the following items are present in the figure legend, table legend, main text, or Methods section.

n/a	Confirmed	
☐	☒	The exact sample size (*n*) for each experimental group/condition, given as a discrete number and unit of measurement
☐	☒	A statement on whether measurements were taken from distinct samples or whether the same sample was measured repeatedly
☐	☒	The statistical test(s) used AND whether they are one- or two-sided<br>*Only common tests should be described solely by name; describe more complex techniques in the Methods section.*
☐	☒	A description of all covariates tested
☐	☒	A description of any assumptions or corrections, such as tests of normality and adjustment for multiple comparisons
☐	☒	A full description of the statistical parameters including central tendency (e.g. means) or other basic estimates (e.g. regression coefficient) AND variation (e.g. standard deviation) or associated estimates of uncertainty (e.g. confidence intervals)
☐	☒	For null hypothesis testing, the test statistic (e.g. *F*, *t*, *r*) with confidence intervals, effect sizes, degrees of freedom and *P* value noted<br>*Give P values as exact values whenever suitable.*
☒	☐	For Bayesian analysis, information on the choice of priors and Markov chain Monte Carlo settings
☒	☐	For hierarchical and complex designs, identification of the appropriate level for tests and full reporting of outcomes
☒	☐	Estimates of effect sizes (e.g. Cohen's *d*, Pearson's *r*), indicating how they were calculated

Our web collection on statistics for biologists contains articles on many of the points above.

Software and code

Policy information about availability of computer code

Data collection	Attune Cytometric software (Thermofischer)<br>BDFACSDIVA (BD Science)<br>IVIS Spectrum imaging system (Caliper Life Sciences, Massachusetts, USA)<br>Bio-Plex® MAGPIX (Bio-Rad)<br>G:box Chemi XX6 device (SYNGENE)<br>EnVision 2104 Multilabel Reader with EnVision Manager 1.12 software (PerkinElmer)<br>EUROLineScan (EUROIMMUN)<br>NIS-Elements F4.30.01 (Nikon)
Data analysis	Graph Pad Prism v8<br>ivingImage software (Caliper Life Sciences)<br>FlowJo version 10 (TreeStar,Ashland, OR) software<br>MaxQuant 2.4.7.0 software<br>Fiji (Image J)<br>MiXCR software package68 (version 4.3.20)<br>R package peptides<br>CLC Main workbench (Version 8.1.3) (QIAGEN Aarhus A/S)

For manuscripts utilizing custom algorithms or software that are central to the research but not yet described in published literature, software must be made available to editors and reviewers. We strongly encourage code deposition in a community repository (e.g. GitHub). See the Nature Portfolio guidelines for submitting code & software for further information.

Data

Policy information about availability of data

All manuscripts must include a data availability statement. This statement should provide the following information, where applicable:

- Accession codes, unique identifiers, or web links for publicly available datasets
- A description of any restrictions on data availability
- For clinical datasets or third party data, please ensure that the statement adheres to our policy

The aligned lymphoma BCR sequences were submitted to Genbank: accession numbers: PV068692 - PV068753 and PV068754 - PV068825. The mass spectrometry data from serum and recombinant Immunoglobulin were submitted to Pride. Accession number: PXD063326

Research involving human participants, their data, or biological material

Policy information about studies with human participants or human data. See also policy information about sex, gender (identity/presentation), and sexual orientation and race, ethnicity and racism.

Reporting on sex and gender	*Use the terms sex (biological attribute) and gender (shaped by social and cultural circumstances) carefully in order to avoid confusing both terms. Indicate if findings apply to only one sex or gender; describe whether sex and gender were considered in study design; whether sex and/or gender was determined based on self-reporting or assigned and methods used. Provide in the source data disaggregated sex and gender data, where this information has been collected, and if consent has been obtained for sharing of individual-level data; provide overall numbers in this Reporting Summary. Please state if this information has not been collected. Report sex- and gender-based analyses where performed, justify reasons for lack of sex- and gender-based analysis.*
Reporting on race, ethnicity, or other socially relevant groupings	*Please specify the socially constructed or socially relevant categorization variable(s) used in your manuscript and explain why they were used. Please note that such variables should not be used as proxies for other socially constructed/relevant variables (for example, race or ethnicity should not be used as a proxy for socioeconomic status). Provide clear definitions of the relevant terms used, how they were provided (by the participants/respondents, the researchers, or third parties), and the method(s) used to classify people into the different categories (e.g. self-report, census or administrative data, social media data, etc.) Please provide details about how you controlled for confounding variables in your analyses.*
Population characteristics	*Describe the covariate-relevant population characteristics of the human research participants (e.g. age, genotypic information, past and current diagnosis and treatment categories). If you filled out the behavioural & social sciences study design questions and have nothing to add here, write "See above."*
Recruitment	*Describe how participants were recruited. Outline any potential self-selection bias or other biases that may be present and how these are likely to impact results.*
Ethics oversight	*Identify the organization(s) that approved the study protocol.*

Note that full information on the approval of the study protocol must also be provided in the manuscript.

Field-specific reporting

Please select the one below that is the best fit for your research. If you are not sure, read the appropriate sections before making your selection.

☒ Life sciences ☐ Behavioural & social sciences ☐ Ecological, evolutionary & environmental sciences

For a reference copy of the document with all sections, see nature.com/documents/nr-reporting-summary-flat.pdf

Life sciences study design

All studies must disclose on these points even when the disclosure is negative.

Sample size	We used cohort sizes of at least 15 mice. 3 such cohorts were analysed. At the start of the experiment, we had no idea about how many mice would develop autoimmunity and lymphoma. We made sure that each cohort was fairly sex matched.
Data exclusions	For serum cytokine analysis, only the mice that later developed B lymphomas were included. Mice that did not develop lymphomas, or developed T lymphomas, were excluded.
Replication	Three independent cohorts were set up to study the development of autoimmunity and lymphomas. The in vitro experiments were done with technical duplicates and repeated at least twice. The in vitro proliferation were done with cells from atl east three different lymphoma mice.
Randomization	For the first two cohorts, breeding was set up with Vλ2315m+/- mice as mothers and TCR-TG+/- mice as fathers. In the third cohort, breeding was set up with TCR-TG+/- mice as mothers and Vλ2315m+/- mice as fathers.
Blinding	There was no blinded group allocation.

Reporting for specific materials, systems and methods

We require information from authors about some types of materials, experimental systems and methods used in many studies. Here, indicate whether each material, system or method listed is relevant to your study. If you are not sure if a list item applies to your research, read the appropriate section before selecting a response.

Materials & experimental systems

n/a	Involved in the study
☐	☒ Antibodies
☐	☒ Eukaryotic cell lines
☒	☐ Palaeontology and archaeology
☐	☒ Animals and other organisms
☒	☐ Clinical data
☒	☐ Dual use research of concern
☒	☐ Plants

Methods

n/a	Involved in the study
☒	☐ ChIP-seq
☐	☒ Flow cytometry
☒	☐ MRI-based neuroimaging

Antibodies

Antibodies used

Flowcytometry:
Single cell suspensions were stained by a number of different fluorochrome-labeled mAbs: Anti-BrdU APC from kit (552598, BD Biosciences, 1:100), Anti-BrdU APC (Bu20a, Biolegend, 1:20) anti-CD19 PE-Cy7 (6D5, Biolegend, 2.5 µg/ml), anti-κ-BV421(RMK-45, Biolegend, 2.5 µg/ml), anti-κ-FITC (187.1, Thermo Fisher Scientific, 8 µg/ml), anti- λ-APC (RML-42, Biolegend, 4 µg/ml), anti-CD80-APC (16-10A1, Biolegend, 8 µg/ml), anti-CD86-PE (GL1, Southern Biotech, 4 µg/ml), anti-mouse I-A/I-E-FITC (2G9, BD Biosciences, 8 µg/ ml), anti-Ki-67-PerCP-Vio700 (REA 183, Miltenyi Biotec, 2 µl/test), anti-CD95-FITC (Jo2, BD Biosciences, 8 µg/ml), anti-CD95-BV605 (SA367H8, Biolegend, 2.5 µg/ml), anti-GL7-FITC (GL7, Biolegend, 8 µg/ml), Biotin-TCRm (anti-pId:MHCII scFv TCR mimetic, homemade, 1.5ug/ml)(Huszthy, Gopalakrishnan et al. 2019), anti-CD4-APC (GK1.5, Southern Biotech, 4 µg/ml), anti-CD4-AF405 (RM4-5, Thermo Fischer Scientific, 4 µg/ml), anti-CD69-PerCP/Cy5.5 (H1.2F3, Biolegend, 3 µg/ml), anti-mouse TCRvβ 8.1-3-FITC (F23.1, Santa Cruz Biotechnology, 1:5) , anti-Id-specific TCR mAb GB113 (Bogen, Gleditsch et al. 1992) (homemade, 8µg/ml), anti-CD93-PE (AA4.1, Biolegend, 3 µg/ml), anti-CD21/CD35-FITC (7G6, BD Biosciences, 8 µg/ml), anti-FoxP3-APC (FJK-16s, Thermo Fisher Scientific, 8 µg/ml) anti-IgM-APC (RMM-1, Biolegend, 8 µg/ml), anti-CD23-PE-Cy7 (B3B4, Thermo Fischer Scientific, 2 µg/ml), anti-CD44-PE-Cy7 (IM7, Biolegend, 3 µg/ml), anti-PD-1-APC-Cy7 (29F.1A12, Biolegend, 1.5 µg/ml), anti-CXCR5-BV421 (L138D7, Biolegend, 6 µg/ml) Goat anti mouse-IgG1-AF488 (polyclonal, Thermo Fisher Scientific, 2 µg/ml), Goat anti mouse-IgG2a (polyclonal, Thermo Fischer Scientific, 2 µg/ml), anti-IgG2b (G15-337, BD Biosciences, 2 µg/ml), anti-CD45R-PerCP/Cy5.5 (RA3-6B2, BD Biosciences, 4 µg/ ml), anti-λ2/3-biotin (2B6, homemade, 6µg/ml)(Bogen 1989), anti-κ-biotin (187.1, homemade, 3µg/ml). The following secondary reagents were used in combination with biotinylated antibodies: streptavidin-APC/Cy7 (405208, Biolegend, 1:300), streptavidin-PerCP (554064, BD Biosciences, 1:500) and streptavidin-PE (554061, BD Biosciences, 1:1000).

Flowcytometry:
Anti-BrdU APC (clone Bu20a, Biolegend, catalogue #339808, 1:20), anti-CD19 PE-Cy7 (6D5, Biolegend, #115520, 2.5 µg/ml), anti-κ-APC-Cy7 (RMK-45, Biolegend, # 409504, 2.5 µg/ml), anti-κ-FITC (187.1, Thermo Fisher Scientific, #OB1170-02, 8 µg/ml), anti-λ-APC (RML-42, Biolegend, #407306, 4 µg/ml), anti-CD80-APC (16-10A1, Biolegend, #104714, 8 µg/ml), anti-CD86-PE (GL1, Southern Biotech, # 735-09L, 4 µg/ml), anti-mouse I-A/I-E-FITC (2G9, BD Biosciences, #553623, 8 µg/ml), anti-Ki-67-PerCP-Vio700 (REA 183, Miltenyi Biotec, #130-120-418, 2 µl/test), anti-CD95-FITC (Jo2, BD Biosciences, #554257, 8 µg/ml), anti-CD95-BV605 (SA367H8, Biolegend, #152612, 2.5 µg/ml), anti-GL7-FITC (GL7, Biolegend, #144604, 8 µg/ml), TCRm-biotin (anti-pId:MHCII scFv TCR mimetic,) (Huszthy, Gopalakrishnan et al. 2019) (homemade, 1.5ug/ml), anti-CD4-APC (GK1.5, Southern Biotech, #1540-11, 4 µg/ml), anti-CD4-AF405 (RM4-5, Thermo Fischer Scientific, #MCD0426, 4 µg/ml), anti-CD69-PerCP/Cy5.5 (H1.2F3, Biolegend, #104520, 3 µg/ml), anti-mouse TCRvβ 8.1-3-FITC (F23.1, Santa Cruz Biotechnology, #sc-33648, 1:5), anti-Id-specific TCR mAb GB113(Bogen, Gleditsch et al. 1992) (homemade, 8µg/ml), anti-CD93-PE (AA4.1, Biolegend, #136504, 3 µg/ml), anti-CD21/CD35-FITC (7G6, BD Biosciences, #553818, 8 µg/ml), anti-FoxP3-APC (FJK-16s, Thermo Fisher Scientific, #17-5773-82, 8 µg/ml) anti-IgM-APC (RMM-1, Biolegend, #406509, 8 µg/ml), anti-CD23-PE-Cy7 (B3B4, Thermo Fisher Scientific, #25-0232-82, 2 µg/ml), anti-CD44-PE-Cy7 (IM7, Biolegend, #103030, 3 µg/ml), anti-PD-1-APC-Cy7 (29F.1A12, Biolegend, #135224, 1.5 µg/ml), anti-CXCR5-BV421 (L138D7, Biolegend, #145512, 6 µg/ml), goat anti mouse-IgG1-AF488 (polyclonal, Thermo Fisher Scientific, #A-21121, 2 µg/ml), goat anti mouse-IgG2a (polyclonal, Thermo Fischer Scientific, #A-21134, 2 µg/ml), anti-IgG2b (G15-337, BD Biosciences, #553884, 2 µg/ml), anti-CD45R-PerCP/Cy5.5 (RA3-6B2, BD Biosciences, #552771, 4 µg/ml), anti-λ2/3-biotin (2B6, homemade, 6µg/ml)(Bogen 1989), anti-κ-biotin (187.1, homemade, 3µg/ml). The following secondary reagents were used in combination with biotinylated antibodies: streptavidin-APC/Cy7 (#405208, Biolegend, 1:300), streptavidin-PerCP (#554064, BD Biosciences, 1:500) and streptavidin-PE (#554061, BD Biosciences, 1:1000). Ki-67 marker (mAb clone REA183, #130-100-339, Miltenyi Biotec).
Immunohistochemistry:
anti-CD3 mAb (clone CD3-12, Bio-Rad, # MCA1477T, 1:100), anti-B220 mAb (clone RA3-6B2, BD Biosciences, # 553090, 1:200), anti-B220-FITC mAb (RA3-6B2, TONBO Biosciences, #SKU 35-0452-U025, 1:200), anti-Bcl-6 pAb (MBS9215821, MyBioSource, #MBS9215821, 1:25) and GB113-PE (clonotype-specific for the Id-sp TCR(Bogen, Lauritzsen et al. 1990), Diatec Monoclonals, 1:100), rabbit AF488-coupled anti-fluorescein (#A-11090, Thermo Fisher Scientific, 1:200), goat anti-PE-Texas Red (#ab34734, Abcam, 1:1000), anti-Pax-5 pAb (#LS-C88806-01, LSBio, 1:100), anti-IBA-1 pAb (#hs-234 013, Synaptic Systems GmbH, 1:200) and anti-CD3 pAb (#sc-1127, Santa Cruz Biotechnology, 1:100).
Western blot:
Cλ2/Cλ3 L chain cross-reactive rat mAb 2B6(Bogen 1989) (1µg/ml), Vλ1/Vλ2 cross-reactive rat mAb 9A8(Bogen 1989) (1µg/ml), goat anti-rat IgG Horseradish Peroxidase (HRP) (#31470, Thermo Fisher Scientific, 1:5000). For Immunoprecipitation, 1µg of anti- λ2315 R/

	A (Rabbit pAb)(Weiss and Bogen 1991), anti-κ mAb (H139-52.1, Sothern Biotech, #1180-01, 1:2000). Western blots under non-reducing condition using the biotin labelled mAb: anti-Cλ2/cλ3 (2B6, 1µg/ml), anti-κ (187.1, 1µg/ml), anti-IgG2aa (8.3, BD Biosciences, #553502, 1:1000), followed by streptavidin-HRP conjugate (#7100-05, Southern Biotech, 1:5000).<br>ELISA:<br>Mouse anti-human IgG (clone R10Z8E9, Invitrogen, #MA5-16929, 1µg/ml) and biotin anti-human L chain κ mAb (clone MHK-49, Biolegend, #316504, 1µg/ml) or biotin anti-human L chain λ mAb (clone MHL-38, Biolegend, #316604, 1µg/ml), streptavidin-Alkaline Phosphatase (ALP) conjugate (#RPN1234, Southern Biotech, 1:3000).<br><br>Immunofluorescence:<br>Goat anti-mouse IgG (H+L) Alexa Fluor™ 488 (#A28175, Invitrogen, 1:1000), anti-mouse IgG1 (γ1) PE (#P21129, Invitrogen, 1:500), mouse IgG2a (γ2a) AF568 (#A21134, Invitrogen, 1:2000), anti-mouse IgG1 (γ1) AF488 (#A21121, Invitrogen, 1:1000), mouse IgG2a (γ2b) AF546 (#A21143, Invitrogen, 1:2000) and anti-mouse IgG1 (γ3) AF488 (#A21151, Invitrogen, 1:1000), 1 µg/ml anti-mouse IgG HRP (#405306, Biolegend), biotinylated anti-mouse IgG1a (#553500, BD Biosciences, 1:1000), anti-mouse IgG2aa (#553502, BD Biosciences, 1:1000), anti-mouse IgG2ba (#553393, BD Biosciences, 1:1000) or anti-mouse IgMa (#553515, BD Biosciences, 1:1000), goat anti-mouse IgG HRP (#405306, Biolegend, 1:5000). rat anti-mouse FITCmAb (#MKAPPA01, Thermo Fisher Scientific, 10µg/ml), Fluorescein/Oregon Green Polyclonal Antibody, Alexa Fluor 488 (#A-11090, Thermo Fisher Scientific), goat anti-mouse λ -TRITC pAb (#1060-03, Southern Biotech, 4µg/ml).
Validation	Most of the antibodies used in this studies were previously tested and validated in the lab. The home made antibodies used, such as 9A8, 2B6, anti- κ (187.1), anti-TCR clonotype specific GB113, anti- λ2315 R/A and anti-pId:MHCII scFv TCR mimetic have been published previously.

Eukaryotic cell lines

Policy information about cell lines and Sex and Gender in Research

Cell line source(s)	NT34 cell line were derived from a primary B lymphoma which arose in a Vλ2315m+/-TCR-TG+/- mouse. These cells were transfected with Effluc. GFP and GFP+ cells were sorted to derive NT34.Effluc cell line. NT34.E2 is a subclone of NT34. A20 cells were obtained from ATTC. A20 transfectant F9 was previously made in the lab and published.
Authentication	These cells were authenicated by flow cytometry staining and BCR sequencing.
Mycoplasma contamination	All cell lines were tested negative for mycoplasma.
Commonly misidentified lines (See ICLAC register)	No, these are not commonly misidentified cell lines.

Animals and other research organisms

Policy information about studies involving animals; ARRIVE guidelines recommended for reporting animal research, and Sex and Gender in Research

Laboratory animals	Inbread strains of mice (BALB/c), Vλ2 modified mice (Vλ2315m+/-) and TCR transgenic mice (TCR-TG+/-) (both on a BALB/c background) were used. Offsprings obtained by Vλ2315m+/- and TCR-TG+/- were used in the study.
Wild animals	n.a
Reporting on sex	Age matched and sex matched animals were used in this study. Even though, Vλ2315m+/-TCR-TG+/- mice from both genders developed autoimmunity and lymphoma, we observed the autoantibody titers and disease development is more predominant in females than males.
Field-collected samples	n.a
Ethics oversight	The mouse experiments were approved by the Norwegian Food Safety Authority (ID 12387, 27752). Experimental procedures followed institutional guidelines of the Department of Comparative Medicine, Oslo University Hospital.

Note that full information on the approval of the study protocol must also be provided in the manuscript.

Plants

Seed stocks	*Report on the source of all seed stocks or other plant material used. If applicable, state the seed stock centre and catalogue number. If plant specimens were collected from the field, describe the collection location, date and sampling procedures.*
Novel plant genotypes	*Describe the methods by which all novel plant genotypes were produced. This includes those generated by transgenic approaches, gene editing, chemical/radiation-based mutagenesis and hybridization. For transgenic lines, describe the transformation method, the number of independent lines analyzed and the generation upon which experiments were performed. For gene-edited lines, describe the editor used, the endogenous sequence targeted for editing, the targeting guide RNA sequence (if applicable) and how the editor was applied.*
Authentication	*Describe any authentication procedures for each seed stock used or novel genotype generated. Describe any experiments used to assess the effect of a mutation and, where applicable, how potential secondary effects (e.g. second site T-DNA insertions, mosiacism, off-target gene editing) were examined.*

Flow Cytometry

Plots

Confirm that:

- [x] The axis labels state the marker and fluorochrome used (e.g. CD4-FITC).
- [x] The axis scales are clearly visible. Include numbers along axes only for bottom left plot of group (a 'group' is an analysis of identical markers).
- [x] All plots are contour plots with outliers or pseudocolor plots.
- [x] A numerical value for number of cells or percentage (with statistics) is provided.

Methodology

Sample preparation	The following mice tissues were analyzed: spleen, the thymus and the lymph nodes. Spleens were collected into GentleMACS C tubes (Miltenyi Biotec) with RPMI tissue culture medium supplemented with 1 mg/ml of collagenase type 4 (LS004188, Worthington) and 0.3 mg/ml DNaseI (D5025, Sigma). The spleens were dissociated with a GentleMACS Dissociator (Miltenyi Biotec) and incubated with collagenase/DNase for 10 minutes at 37°C. Single cell suspensions were washed in complete medium and incubated with Tris-Buffered Ammonium Chloride (ACT) for 7 minutes on ice to lyse erythrocytes. Following ACT treatment, the cells were washed again in complete medium and viable cells were counted using a cell counter.
Instrument	Attune NxT flow cytometer (Thermo Fisher Scientific), LSRII (BD Bioscience)
Software	Attune Cytometric software was used to acquire samples in Attune NxT flow cytometer. BDFACSDIVA was used for acquistion in LSRII. Flow cytometry data were analyzed with FlowJo version 10 (TreeStar, Ashland, OR) software.
Cell population abundance	Young Vλ2315m+/-TCR-TG+/- have around 20% TCR transgenic. Id-specific T cells and around 0.5% B cells that express pId:MHCII. In the mice that developed T and B lymphomas, expanisions of Id-specific T cells and pId:MHCII+ B cells were observed. The frequencies of these population varied between individual mice.
Gating strategy	Lymphocytes were gated first, followed by exclusion of doublets and dead cells. Then the T and B cells were gated using T cell specific antibodies (anti-CD4,anti-CD8, anti-CD3) and B cell specific antibody (anti-CD19). The gated B and T cells were further gated for different markers.

- [x] Tick this box to confirm that a figure exemplifying the gating strategy is provided in the Supplementary Information.
